# Continuous positive airway pressure (CPAP) after lung resection: a randomized clinical trial: Pressão positiva contínua nas vias aéreas (CPAP) após ressecção pulmonar: ensaio clínico randomizado

**DOI:** 10.1590/1516-3180.2014.1321525

**Published:** 2014-02-01

**Authors:** Lígia dos Santos Roceto, Fernanda Diório Masi Galhardo, Ivete Alonso Bredda Saad, Ivan Felizardo Contrera Toro

**Affiliations:** I MSc. Physiotherapist (PT), Intensive Care Unit of Clinical Hospital, School of Medical Sciences, Universidade Estadual de Campinas (Unicamp), Campinas, São Paulo, Brazil; II BSc. Physiotherapist (PT), Intensive Care Unit of Clinical Hospital, School of Medical Sciences, Universidade Estadual de Campinas (Unicamp), Campinas, São Paulo, Brazil; III PhD. Physiotherapist (PT), Pulmonary Rehabilitation Department of Clinical Hospital, School of Medical Sciences, Universidade Estadual de Campinas (Unicamp), Campinas, São Paulo, Brazil; IV MD, PhD. Head and Professor of Thoracic Surgery Department of Clinical Hospital, School of Medical Sciences, Universidade Estadual de Campinas (Unicamp), Campinas, São Paulo, Brazil

**Keywords:** Continuous positive airway pressure, Thoracic surgery, Postoperative complications, Physical therapy specialty, Thoracotomy, Positive-pressure respiration, Pressão positiva contínua nas vias aéreas, Cirurgia torácica, Complicações pós-operatórias, Fisioterapia, Toracotomia, Respiração com pressão positiva

## Abstract

**CONTEXT AND OBJECTIVE:**

Noninvasive mechanical ventilation during the postoperative period (PO) following lung resection can restore residual functional capacity, improve oxygenation and spare the inspiratory muscles. The objective of this study was to assess the efficacy of continuous positive airway pressure (CPAP) associated with physiotherapy, compared with physiotherapy alone after lung resection.

**DESIGN AND SETTING:**

Open randomized clinical trial conducted in the clinical hospital of Universidade Estadual de Campinas.

**METHOD:**

Sessions were held in the immediate postoperative period (POi) and on the first and second postoperative days (PO1 and PO2), and the patients were reassessed on the discharge day. CPAP was applied for two hours and the pressure adjustment was set between 7 and 8.5 cmH_2_O. The oxygenation index (OI), Borg scale, pain scale and presence of thoracic drains and air losses were evaluated.

**RESULTS:**

: There was a significant increase in the OI in the CPAP group in the POi compared to the Chest Physiotherapy (CP) group, P = 0.024. In the CP group the OI was significantly lower on PO1 (P = 0,042), than CPAP group. The air losses were significantly greater in the CPAP group in the POi and on PO1 (P = 0.001, P = 0.028), but there was no significant difference between the groups on PO2 and PO3. There was a statistically significant difference between the groups regarding the Borg scale in the POi (P < 0.001), but there were no statistically significant differences between the groups regarding the pain score.

**CONCLUSION:**

CPAP after lung resection is safe and improves oxygenation, without increasing the air losses through the drains. CLINICAL TRIAL REGISTRATION: NCT01285648

## Abstract (pt)

**CONTEXTO E OBJETIVO:**

A ventilação mecânica não invasiva no período pós-operatório (PO) de ressecção pulmonar pode restaurar a capacidade residual funcional, melhorar a oxigenação e poupar os músculos inspiratórios. O objetivo deste estudo foi avaliar a eficácia da CPAP associada à fisioterapia comparada à fisioterapia unicamente após ressecção pulmonar.

**ESTUDO E LOCAL:**

Ensaio clínico randomizado aberto, realizado no Hospital das Clínicas da Universidade Estadual de Campinas.

**MÉTODO:**

Os atendimentos foram realizados nos PO imediato (POi), primeiro e segundo (PO1, PO2) dias, e a reavaliação na alta hospitalar. A CPAP foi aplicada durante duas horas e o ajuste pressórico estabelecido entre 7 e 8,5 cmH_2_O. Foram analisados índice de oxigenação (IO), escala de Borg e de dor, presença e perda aérea dos drenos torácicos.

**RESULTADOS:**

No grupo CPAP ocorreu aumento significativo do IO no POi (P = 0,024), comparado com o grupo fisioterapia respiratória. Houve redução significativa do IO no PO1 (P = 0,042) para o grupo fisioterapia respiratória, comparando-se à CPAP. A perda aérea foi significativamente maior para o grupo CPAP no POi e PO1 (0,001; 0,028), mas nos PO2 e no PO3 não houve diferença significativa entre os grupos. Foi verificada diferença significativa entre os grupos para a escala de Borg no POi (P < 0,001), porém para a escala de dor não foram verificadas diferenças significativas entre os grupos.

**CONCLUSÃO:**

A CPAP após ressecção pulmonar é segura e melhora a oxigenação sem aumentar a perda aérea pelos drenos. REGISTRO DE ENSAIO CLÍNICO: NCT01285648

## INTRODUCTION

Patients in the postoperative period (PO) following lung resection surgery present a high risk of developing pulmonary complications like retention of secretions, atelectasis, pneumonia, prolonged air leaks and respiratory failure, which prolong the duration of mechanical ventilation and hospitalization and contribute towards increasing mortality.^1^ ^-^ ^3^

Continuous positive airway pressure (CPAP) during the postoperative period, using nasal or full face masks, can restore the residual functional capacity to preoperative levels, improve oxygenation, preserve the inspiratory muscles, restore gas exchange and avoid tracheal intubation due to acute respiratory failure in these patients.^3^ ^,^ ^4^ ^-^ ^7^ However, it has not yet been established whether use of CPAP during the immediate postoperative period after lung resection is more beneficial than treatment with chest physiotherapy, or whether the positive pressure can increase or worsen the air leaks.

## OBJECTIVE

To assess the efficacy of CPAP associated with physiotherapy, compared with physiotherapy alone after lung resection, regarding the following outcomes: oxygenation, dyspnea, pain, duration of stay and air leaks from chest tubes during the postoperative period.

## METHODS

### Specifications of the study

This was a prospective, non-blinded, randomized, comparative, interventional clinical trial.

### Subjects

All the patients who were admitted to the hospital with indications for lung resection were assessed in relation to the eligibility criteria. 60 patients aged 40-75 years of both genders were selected between October 2007 and November 2009. All of them had a medical diagnosis of lung cancer and an indication for lobectomy, bilobectomy or pneumonectomy with posterolateral thoracotomy, and had been admitted to the pulmonology ward of a clinical hospital belonging to a public university. After surgery, the patients were randomized with opaque, sealed envelopes. Both the investigator and the patient knew which group the patient was allocated to. The project was approved by the institution's Research Ethics Committee, under number 388/2007, and all subjects who agreed to participate in the study signed a consent form.

### Exclusion criteria

The surgical indication was established by the medical team, and patients with forced expiratory volume in the first second (FEV_1_) that was less than 30% of the predicted value, or presented advanced-stage disease with Karnofsky performance status (KPS)^8^ 3 or 4, were excluded. Patients who refused to participate in the survey, those who were not aged between 40 and 75 years and those who underwent lung resection with incisions other than posterolateral were excluded. Moreover, patients presenting the following contraindications for use of noninvasive ventilation (NIV) were also excluded: hemodynamic instability that was not responsive to vasoactive drugs; psychomotor agitation or inability to cooperate; evidence of pulmonary thromboembolism and asthma; emergency endotracheal intubation; inability to protect the airway (impaired coughing and swallowing); significant abdominal distension; multiple organ failure affecting more than two organs or systems; or inability to tolerate the nasal mask.

### Preoperative evaluation

During the preoperative period (PRE), the subjects who had been selected for the study underwent physiotherapy evaluation that included taking the clinical history, physical examination, pulmonary function tests and arterial blood gas measurement. In addition, during this period, the patients were informed about the surgical procedure, the type of incision, the intubation and sedation procedures and risks, the importance of coughing during the postoperative period and the need for commitment to the required confinement to bed. The patients were also administered chest physiotherapy comprising incentive spirometry and ventilatory patterns.

### Surgical procedures

Muscle-sparing thoracotomy with no sectioning of the *latissimus dorsi* and *serratus* muscles was performed on all the patients. Epidural anesthesia, as well as regular pain killers, anti-inflammatories and morphine were administered on the first PO. Fissures were treated with cautery dissection and stapling. All the patients received two 38F chest tubes, except in cases of pneumonectomy, which were not drained. The operation was performed by the same surgeon, and the patients were extubated in the operating room.

After surgery, during the immediate postoperative period (POi) and two to four hours after weaning from invasive ventilation and extubation, the patient was again evaluated and was allocated to one of the following two groups:

*Chest physiotherapy*

Chest physiotherapy (CP) was started with one session in the POi and two sessions on the first and second days after surgery (PO1 and PO2). CP consisted of bronchial hygiene techniques and pulmonary expansion, in addition to exercises, and the patients received oxygen supplementation to maintain pulse oximetry saturations higher than 90%. The bronchial hygiene techniques used included forced expiration, coughing and vibration.^9^ Incentive spirometry and breathing patterns associated with movements of the upper and lower limbs were used to maximize deep diaphragmatic breathing.^10^ ^,^ ^11^ The use of bronchodilators and analgesia complied with the standardization and medical indications of the institution's post-anesthesia intensive care unit (ICU).

*CPAP*

This group combined chest physiotherapy with NIV via nasal masks for two hours, using the Nellcor/Puritan Bennett GoodKnight 420G CPAP system (United States). The pressure was adjusted according to the patient's tolerance. The starting pressure was between 7 cmH_2_O and 8.5 cmH_2_O, while the breathing rate was maintained at less than 30 rpm, and the supplemental oxygen was used to maintain pulse oximetry saturation higher than 90%. CPAP was administered from the POi until the second postoperative day, twice a day for a total of five sessions, until reaching 48 to 60 hours after the operation.

### Postoperative evaluation and data gathering

From the POi until hospital discharge, the presence of chest tubes and air leaks was recorded.

After pulmonary function tests had been performed preoperatively, and on hospital discharge, the patients were referred to our hospital's pulmonary function laboratory, where spirometry was performed using the MGC pulmonary function analysis system PC-4000-AM, and anthropometric data were gathered. During the testing, the patient remained seated, using a nose clip, and the percentages of forced vital capacity (FVC) and FEV_1_, and the ratio between them, were determined. Blood samples for arterial blood gas measurements were collected once a day preoperatively, in the POi and on PO1 and PO2. The measurements were made using the Start New Profile 5 ABL-625 and ABL-700 machines, and the ratio between the partial oxygen pressure (PaO_2_) and the inspired oxygen fraction (FiO_2_) was calculated as the oxygenation index (OI).

Before beginning physiotherapeutic protocols, the patients were asked to rate their pain from zero to ten according to its intensity (the larger the score, the greater the intensity of pain). In addition, they were also asked about their sensation of dyspnea according to the Borg scale,^12^ which also ranged from zero to ten.

### Definition of outcomes

The presence of chest tubes and air leaks was verified in the POi and on PO1, PO2 and on the fifth postoperative day or at the time of hospital discharge (PO3). Air leaks were ascertained before beginning the protocol treatment, by watching for one minute to see whether there were any air leaks in the water seal. It was determined whether drain use should continue, or whether the drains should be removed or should be used with wall suction, by applying the institution's medical protocol, through analysis on chest radiographs and on the amount of drained fluid. Thus, it was defined that the drain would be removed when the drainage rate was less than or equal to 200 ml over a 24-hour period.

### Statistical analysis

To describe the profile of the sample according to the study variables, frequency tables containing the absolute frequencies (n) and percentages (%) were calculated for the categorical variables, along with descriptive statistics on the continuous variables with means and standard deviations. 

All patients admitted to our hospital between October 2007 and November 2009 who conformed to the inclusion criteria were selected for the study. Thus, no sample size calculation was performed and the study subjects constituted a convenience sample. It was found that, considering the OI variable, the number of patients was at least five per group (which would provide a power of 80.0%). The mean sample size therefore provided a power of 87.1%.

To compare the categorical variables between the groups, the chi-square test and Fisher's exact test were used, and for continuous variables compared between pairs of groups at baseline, the Mann-Whitney test was used. To compare measurements between longitudinal groups, we used analysis of variance for repeated measurements (repeated-measure ANOVA), always followed by the Tukey multiple-comparison test for groups. Profile tests with contrasts were used to examine the evolution of the assessments in each group. 

Per-protocol (efficacy) analysis was used to assess the outcomes. The variables were transformed into ranks due to lack of normal distribution. The significance level for the statistical tests was 5% (P < 0.05).

## RESULTS

**Figure 1:**
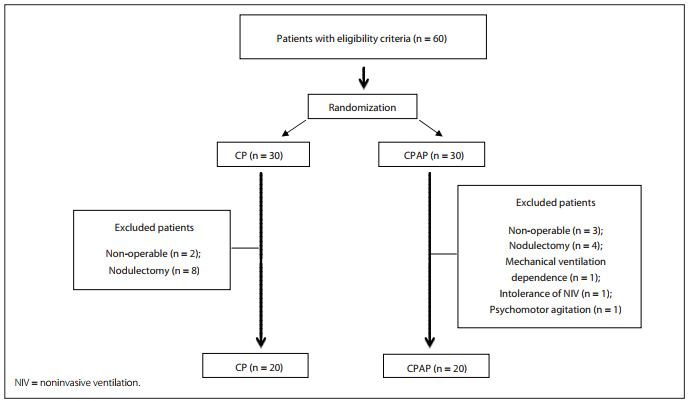
Study flow chart for inclusion and exclusion of patients, for the chest physiotherapy group (CP) and continuous positive airway pressure group (CPAP).

Forty patients were included in this study: 20 in the CP group, which consisted of 10 males and 10 females; and 20 in the combined CP and CPAP group, which contained 7 females and 13 males. Twenty patients were excluded: 12 underwent nodulectomy; 5 were found to be non-operable during surgery; and there was 1 case each of dependence on mechanical ventilation during the postoperative period; intolerance to NIV; and psychomotor agitation (Figure 1). There were 28 cases of lobectomy, 10 of pneumonectomy and 2 of bilobectomy, and the average CPAP pressure used was 7.85 ± 0.4 cmH_2_O. Table 1 shows the analysis on the two groups, with the patients' preoperative characteristics in terms of age, smoking history, body mass index and ventilatory parameters.

**Table 1:**
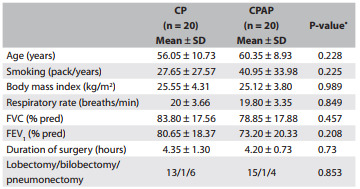
Analysis on the preoperative characteristics of the chest physiotherapy group (CP) and continuous positive airway pressure group (CPAP), with regard to age, smoking history, body mass index and ventilatory parameters

FVC = forced vital capacity; FEV1 = forced expiratory volume in one second; % pred = percentage of the predicted value. Data are mean ± standard deviation (SD). ^*^P-values refer to between-group comparisons.

### Oxygenation index (OI)

**Figure 2:**
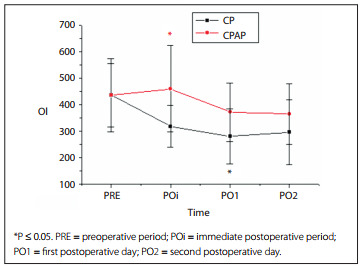
Graphical representation of the evolution of the oxygenation index (OI) in the preoperative period and immediate postoperative period, and on the first and second postoperative days, for the chest physiotherapy group (CP) and continuous positive airway pressure group (CPAP).

The evolution of OI in the two intervention groups is shown in Figure 2, with regard to the preoperative period, immediate postoperative period (POi) and the first and second postoperative days (PO1 and PO2). In the CP group, the OI was significantly lower on PO1 (P = 0.042) than in the CPAP group. The OI in the CPAP group was significantly higher in the POi than the OI in the CP group (P = 0.024).

In a specific analysis on OI with regard to pneumonectomy, there was no significant difference between the groups (P = 0.051; P = 0.0807; P = 0.086), respectively for the times PRE, POi and PO1.

### Air leaks

**Figure 3:**
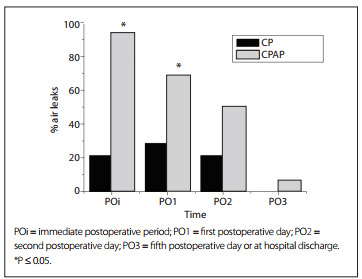
Percentages of patients with air leaks in the chest physiotherapy (CP) group and continuous positive airway pressure (CPAP) group.

The chest tube drainage was analyzed in relation to the 30 patients who underwent lobectomy or bilobectomy; drainage was not performed after pneumonectomy. There were higher air leaks in the CPAP group in the POi and on PO1, than on the CP group (P = 0.001 and P = 0.028, respectively), but on PO2 and on the fifth postoperative day or at hospital discharge (PO3), there was no significant difference in air leaks between the groups (P = 0.105 and P = 1) (Figure 3).

### Dyspnea scale

**Figure 4:**
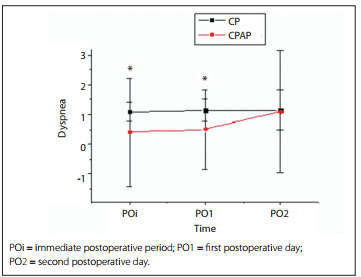
Graphical representation of the incidence of dyspnea based on the Borg scale in the immediate postoperative period and on the first and second postoperative days, in the chest physiotherapy group (CP) and continuous positive airway pressure group (CPAP).

During the preoperative period, 70% of the patients in the CP group did not report dyspnea, whereas 5%, 10% and 15% reported dyspnea when making small, medium and high efforts respectively. In the CPAP group, 60% of the subjects said that they did not have dyspnea, 10% reported dyspnea on making moderate effort and 30% reported dyspnea after great effort, without any statistical difference between the groups (P = 0.704). Analysis on the dyspnea in the POi and on PO1 showed significant differences between the groups (P < 0.001), as can be seen in Figure 4. 

### Pain scale

There were no significant differences between the two intervention groups in terms of the analogue pain scale or the presence of epidural catheters for analgesia (ECA) at the three times (Table 2). 

**Table 2:**
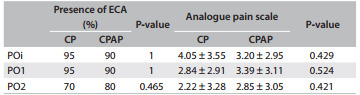
Percentages of patients with epidural catheters for analgesia (ECA) and analogue pain scale scores, in the chest physiotherapy (CP) group and the combination CP and continuous positive airway pressure (CPAP) group

POi = immediate postoperative period; PO1 = first postoperative day; PO2 = second postoperative day

## DISCUSSION

The results presented in this study, including improved OI, no increase in air leaks with positive pressure during the postoperative period and no increases on the pain scale or in relation to dyspnea showed that preventive application of CPAP during the immediate postoperative period after lung resection is safe and is based on the physiological effects of noninvasive ventilation. Likewise, it improved gas exchange, reversed atelectasis and enhanced the distribution of ventilation through recruitment of collapsed areas.^3^ ^,^ ^6^ ^,^ ^7^ ^,^ ^13^ These aspects of lung function are directly related to respiratory complications during the postoperative period.

Use of NIV may have provided improved OI in these patients, which was also found in other studies^3^ ^,^ ^6^ ^,^ ^7^ ^,^ ^14^ because CPAP ventilation mode generates continuous positive airway pressure during inspiration and expiration that prevents alveolar collapse and atelectasis, maintains residual functional capacity and reduces the burden on the left ventricle through improved cardiac function. The studies by Battisti et al. and Kindgen-Milles et al.^5^ ^,^ ^15^ used CPAP with lower pressures, like in the present study, in patients with non-hypercapnic respiratory failure during the postoperative period after lung resection and thoracoabdominal surgery, respectively, and showed that the OI increased after application of NIV, and that there were fewer pulmonary complications and shorter hospital stays.^13^ Moreover, application of CPAP can reduce the respiratory load through an increase in the intrinsic positive end-expiratory pressure (PEEP), which was generated by balancing the load imposed during inspiration.^13^ ^,^ ^16^ The main consequences of high lung volumes during surgery are cell damage caused by over distension and shear forces, and compromised gas exchange, as shown by the oxygenation index.^17^

Pneumonectomy involves greater resection of lung parenchyma and consequently greater impairment of lung function.^18^ In the specific OI analysis on pneumonectomy in the present study, it was observed that there was no significant difference between the groups at the times PRE, POi and PO1. It was found that, considering the OI variable to be a primary outcome of the present study, the number of patients was at least five per group (which would provide a power of 80.0%). The mean sample size therefore provided a power of 87.1%, which justified the lack of sample size calculation. Thus, the study subjects constituted a convenience sample. 

Comparison of air leaks between two groups showed that a higher percentage of the patients with air leaks were receiving positive pressure. However, this difference was only significant until the first postoperative day. In other words, use of NIV did not cause increased air leaks or fistulas. This was shown in another study by air leak and air drainage times that extended beyond seven days.^19^ The study by Lefebvre et al.^14^ found that only one of the 89 patients who received NIV after suffering respiratory failure postoperatively subsequent to lung resection presented persistent air leaks, and this result was attributed to application of low pressure, which was used in our study. Other studies have been consistent with our findings, such as the study by Perrin et al.,^3^ in which no difference in the duration of chest tube drainage between groups with conventional treatment and NIV was shown, even with use of higher inspiratory pressures during application of bilevel ventilation. Use of positive pressure exerts a force on the suture, increases the spring mechanism and further increases the tendency towards distancing from skin edges.^20^ However, other studies have shown that prolonged air leaks after lung resection are related to a number of risk factors, including impairment of lung function, fragility of the lung parenchyma, steroid use, operative protocols for upper lobectomy and presence of pleural adhesions.^19^ ^,^ ^21^ ^,^ ^22^ In the analysis on air leaks, it could be inferred that the evaluation method was subjective, since no researcher had any device that would numerically quantify air leaks. Instead, they just made observations per minute.

With regard to dyspnea, the results showed that the CP group had higher values on the Borg scale than shown CPAP. However, these findings did not have clinical relevance because the data were correlated with degrees of dyspnea that were defined as very mild or absent. In the analysis on the analogue pain scale, there was no significant difference between the values, but they decreased after the operation and this may have been related to removal of the drains. The study by Lima et al.^23^ evaluated the influence of the thoracic drain on the reported pain and found that drain removal resulted in decreased analogue pain scores, which suggests that presence of a chest tube is an important factor associated with pain and functional limitations.

The present study had limitations relating to the public institution where the research was performed, in that there were financial constraints on purchasing and maintaining the equipment that was used in the study. Moreover, mortality rates, infection, reintubation and systemic and local complications during the postoperative period were not analyzed, since hospital length of stay was not verified because of organizational conditions and varied distribution of ICU beds and places for medical specialties. Further investigation on this issue is needed, with larger numbers of patients and blinded evaluators. It is difficult to eliminate bias when a study and its investigators cannot be blinded such that any conscious or unconscious interference in the results from an experiment are avoided. Through other studies in the future, it might be possible to identify the NIV pressure limits, minimum and maximum time of application and long-term outcomes after introduction of NIV during the postoperative period.

There is growing understanding of physiotherapeutic interventions during the postoperative period following lung resection. The recommendations in the literature^4^ ^,^ ^13^ ^,^ ^24^ ^-^ ^26^ highlight the importance of using incentive spirometers and applying chest physiotherapy with regard to reducing costs, hospital length of stay and incidence of atelectasis.^24^ The present study was able to show that use of NIV in thoracic surgery is safe when applied by trained professionals, and showed the need for further research with larger numbers of patients, in order to determination of other benefits and advantages of NIV during the postoperative period following lung resection.

## CONCLUSIONS

Similar to CP, preventive application of CPAP during the postoperative period after lung resection was shown in our study to be a safe technique that was effective in improving oxygenation without increasing air leaks through the thoracic drains. However, further studies with blind assessment that take other relevant outcomes into consideration and include larger numbers of patients are still necessary. 
